# Variation in the location of the shoe sole flexion point influences plantar loading patterns during gait

**DOI:** 10.1186/1757-1146-7-20

**Published:** 2014-03-19

**Authors:** Babette C van der Zwaard, Benedicte Vanwanseele, Fred Holtkamp, Henriëtte E van der Horst, Petra JM Elders, Hylton B Menz

**Affiliations:** 1 EMGO + Institute for health and care research, department of general practice and elderly care medicine, VU University Medical Centre, Postbus 7057, 1007 MB Amsterdam, The Netherlands; 2 Lectorate Health Innovations and Technology, FontysUniversity for Applied Sciences, Eindhoven, The Netherlands; 3 Department of Kinesiology, KULeuven, Leuven, Belgium; 4 Lower Extremity and Gait Studies Program, Faculty of Health Sciences, La Trobe University, Bundoora, Australia

## Abstract

**Background:**

Several footwear design characteristics are known to have detrimental effects on the foot. However, one characteristic that has received relatively little attention is the point where the sole flexes in the sagittal plane. Several footwear assessment forms assume that this should ideally be located directly under the metarsophalangeal joints (MTPJs), but this has not been directly evaluated. The aim of this study was therefore to assess the influence on plantar loading of different locations of the shoe sole flexion point.

**Method:**

Twenty-one asymptomatic females with normal foot posture participated. Standardised shoes were incised directly underneath the metatarsophalangeal joints, proximal to the MTPJs or underneath the midfoot. The participants walked in a randomised sequence of the three shoes whilst plantar loading patterns were obtained using the Pedar® in-shoe pressure measurement system. The foot was divided into nine anatomically important masks, and peak pressure (PP), contact time (CT) and pressure time integral (PTI) were determined. A ratio of PP and PTI between MTPJ2-3/MTPJ1 was also calculated.

**Results:**

Wearing the shoe with the sole flexion point located proximal to the MTPJs resulted in increased PP under MTPJ 4–5 (6.2%) and decreased PP under the medial midfoot compared to the sub-MTPJ flexion point (−8.4%). Wearing the shoe with the sole flexion point located under the midfoot resulted in decreased PP, CT and PTI in the medial and lateral hindfoot (PP: −4.2% and −5.1%, CT: −3.4% and −6.6%, PTI: −6.9% and −5.7%) and medial midfoot (PP: −5.9% CT: −2.9% PTI: −12.2%) compared to the other two shoes.

**Conclusion:**

The findings of this study indicate that the location of the sole flexion point of the shoe influences plantar loading patterns during gait. Specifically, shoes with a sole flexion point located under the midfoot significantly decrease the magnitude and duration of loading under the midfoot and hindfoot, which may be indicative of an earlier heel lift.

## Background

Wearing shoes is an inherent part of our daily lives; however research suggests that some footwear characteristics such as high heels and ill-fitting shoes can have detrimental effects on the foot. Wearing high-heeled shoes has been shown to increase plantar pressure and ground reaction force [1,2], increase the risk of falling [3,4] and change spatial gait characteristics [5]. Wearing ill-fitting shoes has been shown to be associated with foot pain, hallux valgus, deformities of the lesser toes, calluses, corns and ulceration [1,6,7]. Shoes have also been shown to restrict the range of motion of the foot in both adults [8] and children [9] although the long term effects of these changes are unclear.

In recognition of the association between footwear characteristics and foot problems, several footwear assessment forms have been developed to assist with the optimum selection and fit of footwear. Although these tools are based on available evidence, they also recommend shoe characteristics based on clinical experience that are currently unverified [10,11]. One of these characteristics is the position of the sole flexion point in the sagittal plane relative to the metatarsophalangeal joints (MTPJs). It is assumed that the sole flexion point should ideally be located directly underneath the MTPJs and that more proximally-located sole flexion points are detrimental [10-12], however this assumption has not yet been evaluated.

Therefore, the aim of this study was to evaluate the effects of three different positions of the sole flexion point on plantar loading during gait: a sole flexion point right underneath the MTPJs (control), one proximal to the MTPJs and one underneath the midfoot. We hypothesised that the more proximally-located sole flexion points would (i) increase the pressure–time integral of the forefoot due to premature heel elevation, and (ii) cause a lateral shift in loading across the MTPJs due to changes in the windlass mechanism during propulsion.

## Method

### Participants

Female staff and students between the age of 20 and 40 were recruited at the Fontys University for Applied Sciences via e-mail. All participants had to have a shoe size (European) between 38 and 41. Participants were excluded if they (i) had a Foot Posture Index outside the normal range (<0 - >6) [13,14], (ii) had rheumatoid arthritis, (iii) had diabetic neuropathy or (iv) were wearing custom made orthotic devices. The Medical Ethics Committee of the Vrije Universiteit Medical Centre approved the study (2009/267) and informed consent was obtained from all participants.

### Footwear conditions

**Figure 1:**
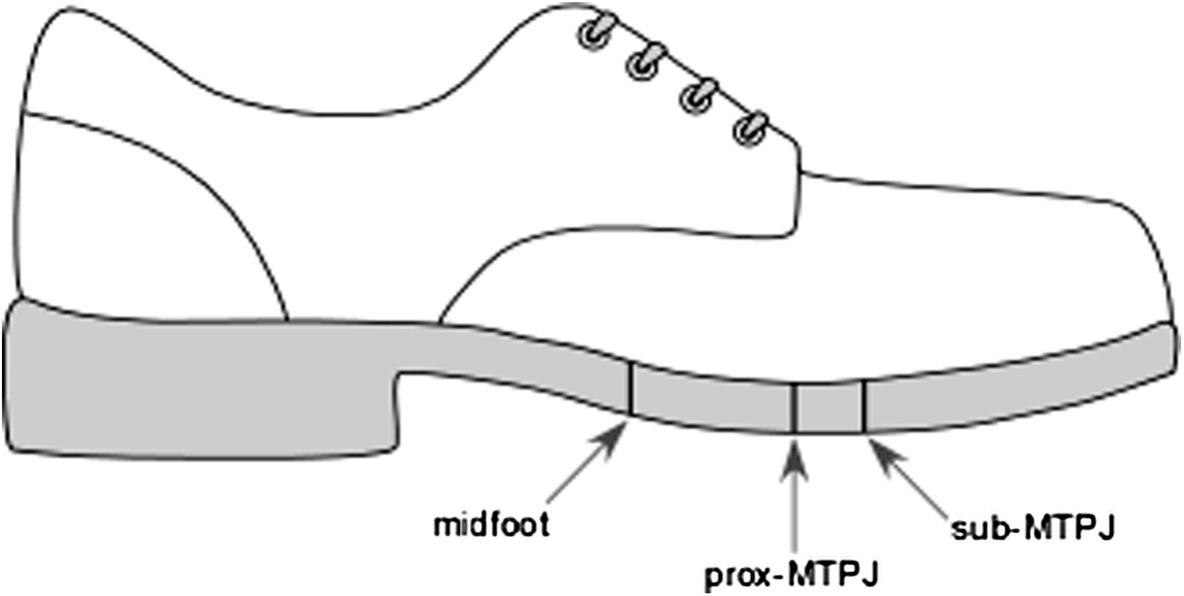
**Incision sites of the soles in order to create differences in sole flexion points.***Sub-MTPJ*: directly underneath the MTPJs, *Prox-MTPJ*: 2–3 cm proximal to MTPJs depending on shoe size, *Midfoot*: underneath tarso-metatarsal joints.

Three shoes (Bata Industrials^©^ type EVA: laced work shoes, nubuck leather upper, PU-sole) with different positions of the sole flexion point were worn by all participants. To create differences in the sole flexion point, the outer soles of the shoes were incised over the full width of the shoe up to the inner sole. Although we acknowledge that other aspects of shoe structure could influence the sole flexion point (such as sole hardness and the flexibility of the upper), the incision approach enabled us to standardise the shoes as much as possible while retaining the overall integrity of the shoe. The tread pattern is scaled proportionally to shoe size. The location of MTPJ 1 was palpated for four people, two in the smallest size and two in the largest size. The location of MTPJ 1 in respect to the outer sole tread pattern was assessed. The chosen orientation of the incision was a straight line similar to the orientation of the axis that combines motion of the MTP 1 and 2 joint. One shoe was incised directly underneath the MTPJ 1 and 2 axis (hereafter referred to as sub-MTPJ); one (depending on the shoe size) 2–3 cm proximal to the MTPJs (hereafter referred to as prox-MTPJ) and one at the level of the tarso-metatarsal joints (hereafter referred to as midfoot) (Figure 1). All incisions were first made in the smallest sized shoe and parallel to the orientation of the MTP 1 and 2 joint axis. The location of the incisions for size 38 in respect to the tread pattern was copied for the larger sizes.

Prior to the study, the shoe size of the right foot was established by means of a shoe size calliper (heel to longest toe), and this size was used for both feet regardless of the size of the left foot. The corresponding size insoles for the plantar pressure assessment were placed in the shoe. All shoes were tied by the same researcher in order to diminish the influence of the lace tightness on the outcome as much as possible. Participants were asked to walk in the shoes for two minutes prior to data collection. During data collection participants walked over an eight metre walkway at their own comfortable speed. The order of presentation of the three shoe conditions was randomised.

### Plantar pressure assessment

**Figure 2:**
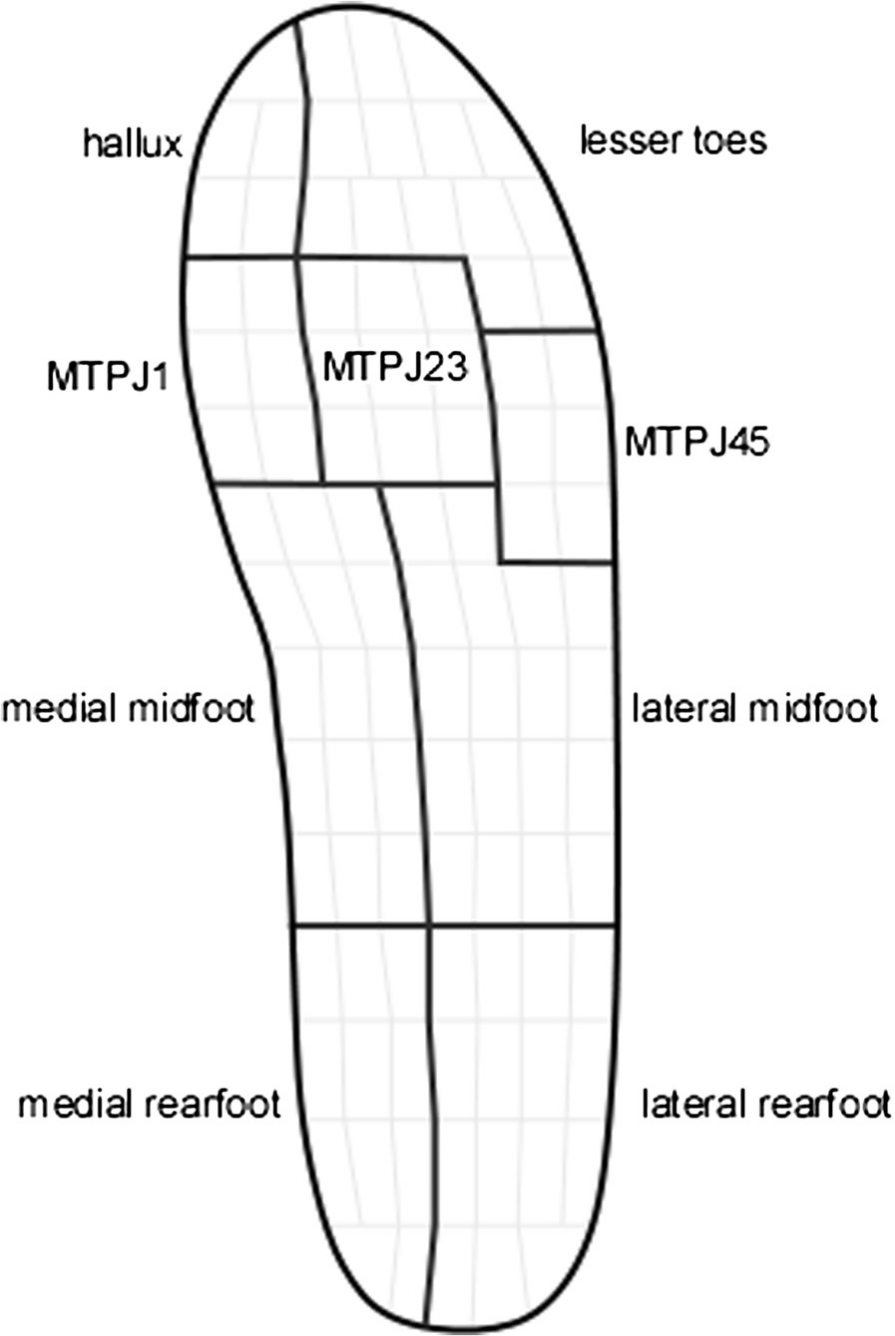
Pedar masks used in plantar pressure measurement.

In-shoe plantar pressures were measured using the Pedar-X system (Novel gmbh, Münich, Germany), which consists of 99 capacitive sensors arranged in a grid and embedded within a thin flexible insole approximately 2 mm thick. A previous study has demonstrated acceptable reliability of this system with the exception of the area under the toes [15]. Data of twelve steps were obtained after excluding initiation, termination and turning steps [16]. Previous findings of Pataky *et al.*[17] highlighted the importance of choosing the edges of masks that are congruent with plantar anatomy. Based on an average peak pressure template of 104 ‘normal and healthy’ feet [18], anatomically correct masks were therefore created to evaluate the pressure-related outcome measures to reflect the more proximal location of MTPJs 4 and 5 and the 4^th^ and 5^th^ toes (Figure 2).

### Outcome measures

Five pressure-related variables were selected *a priori* for analysis: peak pressure (PP), contact time (CT), pressure–time integral (PTI) and the ratio PP and PTI between masks MTPJ1 and MTPJ2-3 (PP and PTI ratio). PP and CT were derived directly from the software. PTI was calculated according the method described by Melai *et al.*[19]:

$$\begin{array}{rl} \mathrm{FTI}÷\mathrm{CA} & (\mathrm{FTI}=\mathrm{Force}\;\mathrm{Time}\;\mathrm{Integral},\\ & \;\mathrm{CA}=\mathrm{Contact}\;\mathrm{Area}) \end{array}$$

The PP and PTI ratio were determined by dividing the PP or PTI of the MTPJ2-3 mask by the PP or PTI of the MTPJ1 mask, to provide an indication of lateral shift in forefoot loading. Walking speed and BMI were measured to test for confounding.

### Statistical analysis

All data were tested for normalcy; the data were defined to be normal distributed when skewness > 1.0 (IBM^©^ SPSS^©^ statistics version 20.0.0). Skewed data were log transformed. For each mask all variables were compared across the three different shoe conditions using multilevel model linear regression (MLwiNversion 2.26 [20]). The different outcome measures were set as level 1, the participants were set as level 2 [21]. Random intercepts and random slopes were added to the basic model and changes of the -2log likelihood ratio were used to evaluate the best model. The confidence interval was set at 95%.

## Results

Twenty-one participants were included for this trial; the participants’ characteristics are shown in Table 1. Both walking speed and BMI were not found to be confounding in any of the data. All results shown (Table 2) are derived from a random intercept model; the addition of a random slope did not improve the model.

**Table 1:** Participant characteristics

	**Average (SD)**	**Range**
Age (years)	27.5 (6.1)	20-39
Height (cm)	171 (6.8)	160-181
BMI	22.9 (3.4)	18-29

**Table 2:** Results

**Peak pressure (PP)**	***Sub-MTPJ***		***Prox-MTPJ***			***Midfoot***		
**(kPa)**	**average**	**(95% CI)**	**average**	**(95% CI)**	**p-value**	**average**	**(95% CI)**	**p-value**
Hallux	278.3	(239.7-316.9)	266.2	(247.4-285.0)	0.208	268.8	(250.0-287.6)	0.322
Lesser toes	112.4	(97.9-126.9)	115	(107.4-122.6)	0.505	114.7	(107.1-122.3)	0.555
MTP 1	244.4	(224.2-264.6)	239.8	(228.2-251.4)	0.436	244.1	(232.5-255.7)	0.959
MTP 2&3	226	(207.4-244.6)	229.9	(222.3-237.5)	0.317	226.1	(218.5-233.7)	0.979
MTP 4&5	145.2	(132.9-157.5)	154.2	(146.2-162.2)	0.028*	147.4	(139.4-155.4)	0.592
Medial midfoot	99.9	(92.6-107.2)	91.5	(86.6-96.4)	0.008*	94	(88.9-99.1)	0.023*
Lateral midfoot	108	(94.5-121.5)	116.2	(101.3-131.1)	0.281	103.1	(88.2-118.0)	0.519
Medial hindfoot	237	(222.5-251.5)	232.9	(224.3-241.5)	0.351	227	(218.4-235.6)	0.023*
Lateral hindfoot	243.3	(228.6-258.0)	239.6	(230.2-249.0)	0.441	231	(221.6-240.4)	0.010*
**Contact time (CT)**	***Sub-MTPJ***		***Prox-MTPJ***			***Midfoot***		
**(ms)**	**average**	**(95% CI)**	**average**	**(95% CI)**	**p-value**	**average**	**(95% CI)**	**p-value**
Hallux	492.5	(461.1-523.9)	495.6	(471.1-520.1)	0.804	493.4	(468.9-517.9)	0.943
Lesser toes	471.8	(440.4-503.2)	497.9	(471.6-524.2)	0.052	486.9	(460.6-513.2)	0.26
MTP 1	511.3	(487.4-535.2)	514.3	(494.7-533.9)	0.764	502.7	(483.1-522.3)	0.9
MTP 2&3	560.3	(538.5-582.1)	570.9	(559.5-582.3)	0.068	570.8	(559.4-582.2)	0.07
MTP 4&5	581.8	(562.0-601.6)	587.7	(577.3-598.1)	0.266	576.4	(565.8-587.0)	0.317
Medial midfoot	608.4	(590.8-626.0)	606.7	(593.0-620.4)	0.808	591	(577.3-604.7)	0.013**
Lateral midfoot	628.7	(611.5-645.9)	624.1	(613.5-634.7)	0.394	607.2	(596.6-617.8)	0.000**
Medial hindfoot	485.7	(449.6-521.8)	472	(439.3-504.7)	0.412	458	(425.3-490.7)	0.097
Lateral hindfoot	444.8	(415.0-474.6)	426.5	(400.8-452.2)	0.162	415.4	(389.7-441.1)	0.025*
**Pressure time integral (PTI)**	***Sub-MTPJ***		***Prox-MTPJ***			***Midfoot***		
**(Ns/cm**^**2**^**)**	**average**	**(95% CI)**	**average**	**(95% CI)**	**p-value**	**average**	**(95% CI)**	**p-value**
Hallux	3.243	(2.735-3.751)	3.238	(3.058-3.418)	0.957	3.243	(3.063-3.423)	1
Lesser toes	1.262	(1.021-1.503)	1.252	(1.152-1.352)	0.845	1.205	(1.105-1.305)	0.264
MTP 1	4.5	(4.035-4.965)	4.452	(4.252-4.652)	0.638	4.405	(4.205-4.605)	0.352
MTP 2&3	4.476	(4.002-4.950)	4.338	(4.171-4.505)	0.105	4.481	(4.314-4.648)	0.953
MTP 4&5	3.181	(2.762-3.600)	3.367	(3.136-3.598)	0.115	3.162	(2.931-3.393)	0.872
Medial midfoot	1.09	(0.955-1.225)	1.09	(1.004-1.176)	1	0.957	(0.871-1.043)	0.003**
Lateral midfoot	1.514	(1.255-1.773)	1.628	(1.371-1.885)	0.384	1.252	(0.995-1.509)	0.046**
Medial hindfoot	3.895	(3.560-4.230)	3.809	(3.631-3.987)	0.345	3.628	(3.450-3.806)	0.003**
Lateral hindfoot	3.852	(3.497-4.207)	3.914	(3.759-4.069)	0.433	3.633	(3.478-3.788)	0.006**
**Ratio’s**	***Sub-MTPJ***		***Prox-MTPJ***			***Midfoot***		
	**average**	**(95% CI)**	**average**	**(95% CI)**	**p-value**	**average**	**(95% CI)**	**p-value**
PP MTP2&3 / MTP 1	0.952	(0.834-1.070)	1	(0.949-1.051)	0.065	0.957	(0.906-1.008)	0.848
PTI MTP2&3 / MTP 1	1.033	(0.898-1.168)	1.081	(1.022-1.140)	0.11	1.09	(1.031-1.149)	0.057

Peak pressure, contact time, pressure–time integral and ratioof MTPJ23/MTPJ1 peak pressure results for each of the three sole flexion points.

*p value < 0.05 compared to sub-MTPJ condition.

**p value < 0.05 compared to sub-MTPJ condition and prox-MTPJ condition.

### Peak pressure

**Figure 3:**
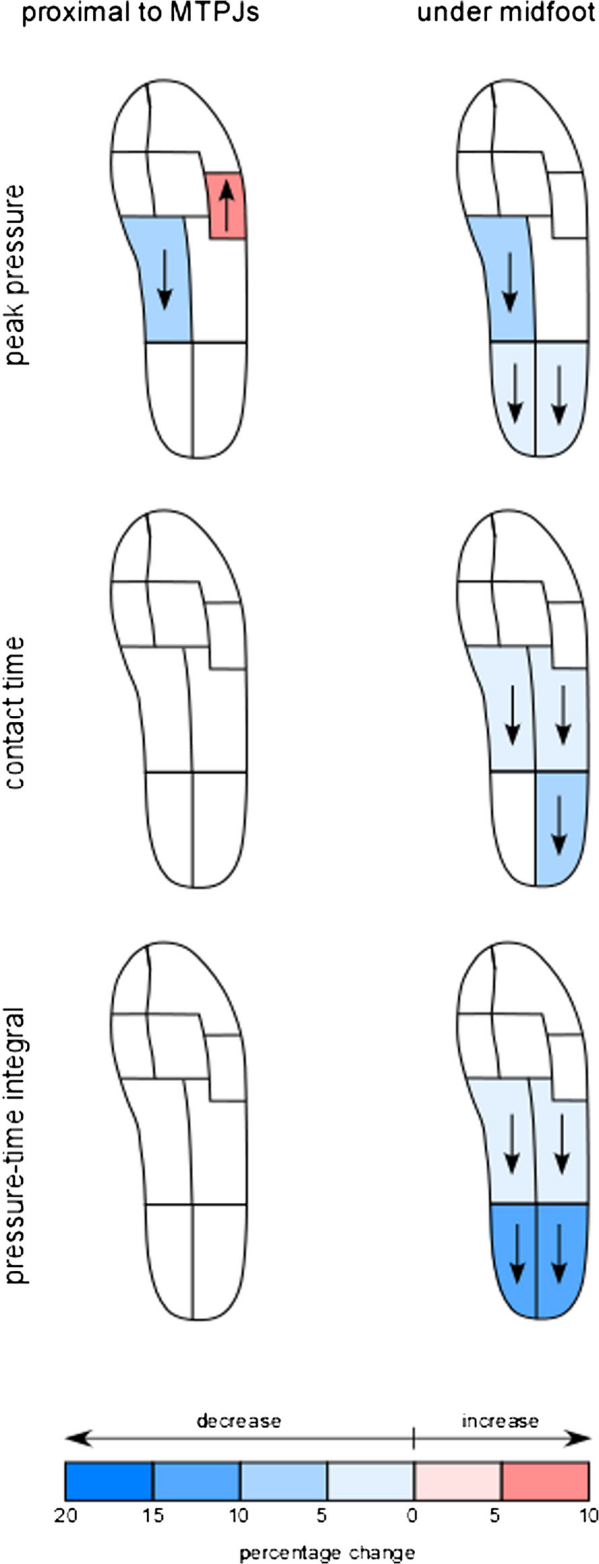
Percentage change in each mask relative to the sub-MTPJ sole flexion point.

Walking with the *prox-MTPJ* versus the *sub-MTPJ* sole flexion point produced changes in PP for two masks (Figure 3). The PP increased in the MTPJ 4–5 mask (avg 154.2 kPa from 145.2 kPa p = 0.028) and decreased in the medial midfoot mask (avg. 91.5 kPa from 99.9 kPa p = 0.008) (Table 1). No other changes were found between these two sole flexion points for any of the other variables. Several differences were found when walking in the shoe with a *midfoot* flexion point. Significant decreases were found in PP medial midfoot (94 kPa p = 0.023), medial hindfoot (227 kPa p = 0.023) and lateral hindfoot (231 kPap = 0.01) when walking with a *midfoot* sole flexion point versus the *sub-MTPJ* sole flexion point (resp. 99.9, 237, 243.3 kPa). No significant changes were found comparing the *midfoot* to the *prox-MTPJ* flexion point.

### Contact time

Contact time was found to decrease in the midfoot (medial: 591 ms p = 0.013 and lateral 607.2 ms p < 0.000) wearing the *midfoot* sole flexion point shoe versus both other flexion points (*sub-MTPJ* resp. 608.4 and 628.7 ms; *prox-MTPJ* resp. 606.7 and 624.1 ms). The lateral hindfoot was found to have a lower CT (415.4 ms p = 0.025) in the *midfoot* shoe versus the *sub-MTPJ* shoe (444.8 ms).

### Pressure time integral

Reduction of the PTI in the medial midfoot (0.957 Ns/cm^2^ p = 0.003), lateral midfoot (1.252 Ns/cm^2^ p = 0.046), medial hindfoot (3.628 Ns/cm^2^ p = 0.003) and lateral hindfoot (3.633 Ns/cm^2^ P = 0.006) were found in the *midfoot* flexion point versus the *sub-MTPJ* sole flexion point (resp. 1.09, 1.514, 3.895 and 3.852 Ns/cm^2^) and the *prox-MTPJ* (resp. 1.09, 1.628, 3.809 and 3.914 Ns/cm^2^).

### Peak pressure and pressure–time integral ratio

Walking in the *midfoot*sole flexion point shoe showed a trend towards a lateral shift in forefoot loading (PTI MTP2-3/MTP1 = 1.09) compared to the *sub-MTPJ* (PTI MTP2-3/MTP1 = 1.03) but this trend was not significant (p = 0.056).

## Discussion

This novel study provides insights into the optimum location of the sole flexion point of the shoe by evaluating the effects of three different positions of the shoe sole flexion point on plantar loading during gait. The current assumption is that a sole flexion point located directly beneath the MTPJs is optimal [10-12]. We found few differences between the shoes with sole flexion points located near the MTPJs (i.e. directly beneath or slightly proximal to the MTPJs). These findings suggest that it is not critical for the sole flexion point to be located *directly* under the MTPJs (as suggested by previous authors [10,11]). Compared to wearing the shoe with the sole flexion point located directly under the MTPJs, wearing the shoe with the sole flexion point proximal to the MTPJs resulted in an increase of peak pressure in the MTP 4–5 area and a decrease in the medial midfoot area. This could suggest that a *prox-MTPJ* situated sole flexion point distributes the plantar pressure marginally better over the entire foot then a *sub-MTPJ* located sole flexion point.

Although few differences were found when comparing the shoes with sole flexion points located near the MTPJs, several differences were found while wearing a sole flexion point located underneath the midfoot. The PP, CT and PTI of both the hindfoot and the midfoot decreased significantly, despite there being no change in walking speed. We therefore postulate that the reduction in magnitude and duration of loading observed in the midfoot and hindfoot may be due to an earlier heel lift. Although the foot is capable of flexing at the tarso-metatarsal joint, it is likely that the heel will also have to lift within the shoe, which could theoretically increase the friction between the posterior aspect of the calcaneus and the heel cup. If this premise is correct, a flexion point underneath the midfoot may be detrimental to foot function. However, this interpretation is inherently speculative, as we did not collect kinematic data to objectively confirm the movement of the foot inside the shoe.

We hypothesized that a proximally-located sole flexion point would transfer the load of the forefoot more laterally (i.e. more towards MTPJs 2 and 3 and less on MTPJ1) as a result of greater lowering of the medial arch. A decrease in arch height would lengthen the distance between forefoot and hindfoot and therefore tension the plantar fascia, leading to decreased mobility of MTPJ1 joint due to the windlass mechanism [22] and a corresponding increase in lateral loading of the MTPJs. This is described by Bojsen-Møller [23] as a “low gear” push off. Although the ratio of PTI MTP2-3/MTP1 was not significant, the p-value of 0.056 does suggest a trend towards a lateral shift with a *midfoot* flexion point. It has been shown that a reduction in PTI under MTPJ2 is correlated to subjective pain improvement in people with forefoot pain [24] and therefore this trend could be clinically important.

The strength of this study is that we used the same type, make and size of shoe and merely incised the sole at different locations to create sole flexion points. As such, any differences observed between the shoes can be confidently attributed to the variation in sole flexion point. However, there are also several limitations to this study. Firstly, we acknowledge that other aspects of shoe structure could influence the sole flexion point, such as sole hardness and the flexibility of the upper. However, the incision approach enabled us to standardise the shoes as much as possible while retaining the overall integrity of the shoe. Secondly, we used pressure measurements to assess the changes in load on the foot. Although increased pressure is an important variable in relation to diabetic foot ulceration [25], the relationship between plantar pressures and pain is inconsistent [26]. Thirdly, we have not assessed kinematics of the foot using motion analysis. Finally, the sample population in this study was homogenous; females between 20 and 40, with a normal foot posture and no foot pain or deformities. Therefore, the results should be interpreted in light of this limitation.

In summary, this study has shown that the location of the sole flexion point of the shoe influences plantar loading patterns during gait. We found few differences between the shoes with sole flexion points located near the MTPJs (i.e. directly beneath or slightly proximal to the MTPJs). These findings suggest that it is not critical for the sole flexion point to be located *directly* under the MTPJs. However, it would appear that a sole flexion point located under the midfoot could detrimentally affect normal foot function, possibly by promoting an earlier heel lift.

## Competing interests

HBM is Editor-in-Chief of *Journal of Foot and Ankle Research*. It is journal policy that editors are removed from the peer review and editorial decision making processes for papers they have co-authored. The other authors declare that they have no competing interests.

## Authors’ contribution

BvdZ was responsible for data-collection and wrote the manuscript. BV and HM assisted with the data evaluation and statistical analysis. BV and HM commented on several drafts of the manuscript. FH, HvdH and PE commented on the first and last draft of the manuscript. All authors have read and approved the final manuscript.
